# Urban Vegetation Mapping from Aerial Imagery Using Explainable AI (XAI)

**DOI:** 10.3390/s21144738

**Published:** 2021-07-11

**Authors:** Arnick Abdollahi, Biswajeet Pradhan

**Affiliations:** 1 Centre for Advanced Modelling and Geospatial Information Systems (CAMGIS), Faculty of Engineering and IT, University of Technology Sydney, Ultimo, NSW 2007, Australia; abolfazl.abdollahi@student.uts.edu.au; 2 Earth Observation Center, Institute of Climate Change, University Kebangsaan Malaysia, Bangi 43600 UKM, Selangor, Malaysia

**Keywords:** XAI, deep neural network, remote sensing, SHAP, vegetation mapping

## Abstract

Urban vegetation mapping is critical in many applications, i.e., preserving biodiversity, maintaining ecological balance, and minimizing the urban heat island effect. It is still challenging to extract accurate vegetation covers from aerial imagery using traditional classification approaches, because urban vegetation categories have complex spatial structures and similar spectral properties. Deep neural networks (DNNs) have shown a significant improvement in remote sensing image classification outcomes during the last few years. These methods are promising in this domain, yet unreliable for various reasons, such as the use of irrelevant descriptor features in the building of the models and lack of quality in the labeled image. Explainable AI (XAI) can help us gain insight into these limits and, as a result, adjust the training dataset and model as needed. Thus, in this work, we explain how an explanation model called Shapley additive explanations (SHAP) can be utilized for interpreting the output of the DNN model that is designed for classifying vegetation covers. We want to not only produce high-quality vegetation maps, but also rank the input parameters and select appropriate features for classification. Therefore, we test our method on vegetation mapping from aerial imagery based on spectral and textural features. Texture features can help overcome the limitations of poor spectral resolution in aerial imagery for vegetation mapping. The model was capable of obtaining an overall accuracy (OA) of 94.44% for vegetation cover mapping. The conclusions derived from SHAP plots demonstrate the high contribution of features, such as Hue, Brightness, GLCM_Dissimilarity, GLCM_Homogeneity, and GLCM_Mean to the output of the proposed model for vegetation mapping. Therefore, the study indicates that existing vegetation mapping strategies based only on spectral characteristics are insufficient to appropriately classify vegetation covers.

## 1. Introduction

In urban areas, vegetation plays a significant role for various purposes, including conserving biodiversity, preserving ecological balance, and reducing the urban heat island effect [1]. Producing these vegetation maps is critical to assist planners in optimizing climate change adaptation and urban ecosystem services [2]. Remote sensing data have been extensively employed for vegetation mapping in various environments due to its ability to discriminate broad scales of land cover types. For vegetation monitoring in urban and rural regions, aerial photography and satellite imaging have been used [3,4,5,6]. Urban vegetation cover is much more fragmented than natural vegetation (i.e., forest, rangeland), making accurate extraction of vegetation cover more complex and challenging. Thus, it has been a hot topic in the remote sensing field to effectively extract and map land use types over urban vegetation cover [7]. Satellite imagery of ultra-high resolution (UHR), unmanned aerial vehicle (UAV) imagery, very-high resolution (VHR), and moderate-to-high-resolution (HR) are employed in urban vegetation mapping [1]. For the high and very high-resolution satellite imagery, QuickBird and IKONOS satellite data have been effectively employed for mapping the urban vegetation cover [7,8]. Moreover, aerial imagery has been broadly utilized in vegetation mapping because the images have better resolution [9]. On the other hand, the fundamental disadvantage of multispectral satellite data with moderate resolution such as Enhanced Thematic Mapper Plus (ETM+) and Landsat Thematic Mapper (TM) is that they are unable to capture sufficient details of ground objects because of its coarse spatial resolution (30 m) [10]. However, using spectral mixture analysis, they still can be utilized to measure vegetation abundance in suburban and urban environments. To track the spatial–temporal changes of urban vegetation, a fraction map can be created [11]. The classification techniques can be classified into two types. First, they can be categorized by object-based image analysis (OBIA) and pixel-based classification methods, which are extensively employed in HR remote sensing image classification and vegetation mapping [12,13]. The former argues that single pixels are autonomous, and they are processed without considering their spatial relationships with other adjacent pixels [14]. However, a “salt and pepper” effect is always apparent in the classification results because the individual pixels no longer take the properties of classification purposes in HR images [15]. OBIA represents the spatial neighborhood attributes rather than an individual pixel, as opposed to pixel-based classification. OBIA’s main characteristic is that it uses multi-scale image segmentation to combine a variety of spatial, spectral, and textural data in the classification process, which greatly improves accuracy [16]. In the processing of VHR images, OBIA categorization approaches have been thoroughly explored, and numerous approaches have been developed to classify objects [17,18,19]. However, during the OBIA process, two important issues remain: selecting appropriate features for image classification and determining the right scale for image segmentation [20]. The OBIA’s performance is mostly dependent on the researchers’ past knowledge and expertise because there is no precise process for optimizing the scale parameter and feature [21]. Furthermore, OBIA necessitates a variety of input variables and image segmentation, making it tough and complex to use, particularly for beginners [3].

Broadly, the classification methods can be classified into unsupervised [22] and supervised [23] classification techniques. Machine learning techniques are commonly used in supervised classification. For instance, Réjichi and Chaâbane [24] created spatial-temporal vegetation maps using the support vector machine (SVM) method, and Wei, et al. [25] used the decision tree to map the vegetation cover. The vegetation map was obtained from Airborne Visible/Infrared Imaging Spectrometer (AVIRIS) data using neural network (NN) by [26]. Tigges, et al. [27] performed an SVM approach to classify urban vegetation using various temporal and spectral band mixtures of five RapidEye imagery for Berlin, Germany. Tooke, Coops, Goodwin, and Voogt [2] applied spectral unmixing and decision tree approach for classifying urban vegetation characteristics from QuickBird images for Vancouver city, Canada. The Iterative Self-Organizing Data Analysis (IOSDATA) technique [28] and K-Means method [29] are examples of unsupervised classification algorithms, which have been popularly used for vegetation cover classification from SPOT-4 and aerial imagery. In [30], two totally unsupervised methods called Jenks Natural Breaks classification method and Agglomerative Hierarchical clustering approach were used for vegetation mapping from IKONOS images for the city of Bamenda, Cameroon. In another work [31], Probabilistic Neural Network (PNN) approach was introduced to classify vegetation covers from SPOT and LANDSAT data for the Marrakech city, Morocco. However, these traditional classification algorithms still need a large amount of reference data, and they are more complex or necessitate more manual involvement for producing accurate classification maps [32]. Moreover, due to the same spectral properties and a complex spatial structure amongst urban vegetation groups, precise vegetation mapping based on the traditional pixel-based classification methods from VHR satellite imagery is still challenging [33]. These approaches, which rely solely on spectral information, had limited effectiveness in urban set-ups [33]. In contrast to pixel-based approaches, the features employed for classification in the object-oriented technique are the objects’ attributes. There are some features reduction methods, which can be used to overcome the limitation of OBIA. For instance, for data reduction and unsupervised feature extraction, principal components analysis (PCA) [34] has been frequently employed. However, it has three major flaws: a significant memory need, a high computational cost, and poor performance when processing massive data sets. In [35], a logistic regression (LR) method was performed for both feature selection based on their relative importance and classification of remote sensing imagery. The assumption of linearity between the dependent and independent variables is a key constraint of LR as well as non-linear problems cannot be solved by LR [35]. Thus, developing an accurate strategy for picking the features that are important for a specific classification task and alleviating the above-mentioned drawbacks is necessary.

Deep neural networks (DNN) have become extremely popular techniques in remote sensing applications, which recognize features in several levels of representation [36]. This is due to the fact that these approaches may easily encode spectral and spatial information from raw images without the need for any preprocessing [37]. They are also a hierarchical structure of deep neural networks and include a number of interconnected layers that may learn a hierarchical feature representation from the data and extract the deep features of the input data [37]. Therefore, we implemented a DNN model in the current work to address the limitation of conventional classification approaches in vegetation mapping from high-resolution remotely sensed imagery. When building a classification model, it is critical to understand why each classification is made, except achieving high accuracy. Due to the complicated architecture of DNNs [38], interpreting the output of these models is typically thought to be extremely difficult [38].

In this work, we illustrate how to interpret the outputs of DNN model designed for classification based on an explanation method. Thus, we present a methodology and evaluation for feature selection based on an explanatory model, which is generated by a method termed Shapley Additive explanations (SHAP) [39,40] to interpret the output of DNN and improve the OBIA limitation in selecting appropriate features for classification. For every feature of every data point, the technique allocates SHAP values, which are contribution values for the output of the model. We utilize the contribution information of every feature to arrange and encode the features based on their importance using these SHAP values. In this scenario, choosing a set of features according to the SHAP values entails sorting the features based on their contributions to the method’s output and then selecting the initial features. We test our method on vegetation mapping from high-resolution remote sensing imagery based on the spectral and textural features. In summary, the following are the main contributions of this study: (1) applying a DNN for producing high-quality vegetation maps from remote sensing data. (2) Implementing the Sharp method as a feature selection mechanism to select appropriate features among the spectral and textural features and see the contribution information of every feature on classification and mapping the vegetation cover. This study is organized in the following way: we give a brief overview of SHAP for selecting features and DNN structure in Section 2. In Section 3 and Section 4, we provide the experiments and discussion on the results, respectively. In Section 5, a concluding remark is presented.

## 2. Methodology

**Figure 1:**
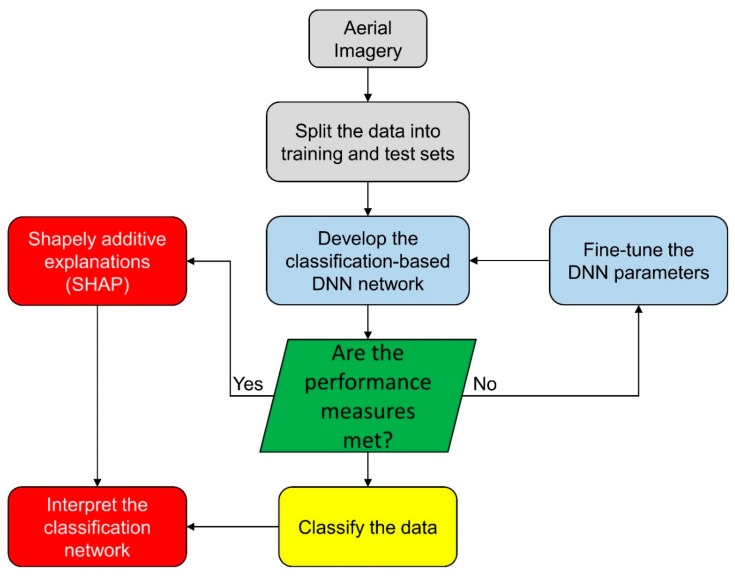
The SHAP workflow for an interpretable DNN model.

The method for finding important variables that influence the classification model is depicted in Figure 1. A random division of the data into training and test sets is used to build the DNN model at first. The training and test dataset are utilized to create the classification model and assess the model’s performance. SHAP is applied to the classification network (in this case, a DNN network) to create additive attributes, which are then utilized to ascertain significance variables and determine the effect of input factors to the classification results. In the following sections, the technique for mapping vegetation covers using DNN and SHAP is described in depth.

### 2.1. Dataset

**Figure 2:**
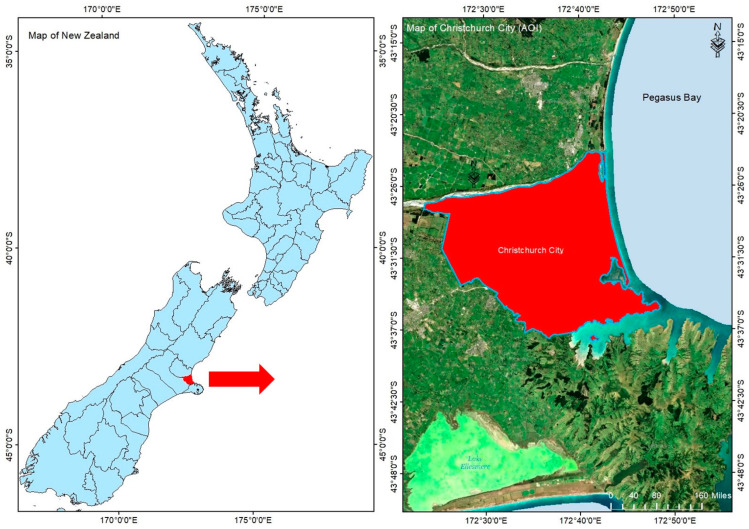
The geolocation of the used AIRS dataset. The AOI is indicated by the red polygon.

We selected three images from the Aerial Imagery for Roof Segmentation (AIRS) [41] dataset with a spatial resolution of 7.5 cm and a dimension of 2500 × 3000 for classification purpose. The test area is a designated area of Christchurch city with 457 km^2^ coverage, which is situated on the South Island of New Zealand (Figure 2) and the images were taken during the flying season of 2016 [41]. Since the aerial images used in this study are RGB images, we used the HSV (hue, saturation, and value) color space of the images instead of RGB. The fundamental distinction is the color representation between both color spaces. The HSV color space uses three parameters named Hue (H), Saturation (S), and Value (V) to depict the color of the object. The H shows the object’s color, while the S and V values represent the object’s color illuminance [42]. This description enables the color to be distinguished from the illumination and prevents the influence of illumination variations on the color of the object. Consequently, the suggested method for vegetation mapping used HSV color space. In addition, to help with the classification process and improve the vegetation classification method [43], we used textural factors based on the gray-level-co-occurrence-matrix (GLCM) [44]. The texture descriptor, GLCM is a statistical matrix of joint conditional possibility distributions between the grey levels of image pixel pairs with a specific distance and direction within a given frame. We utilized eCognition Developer 64 software to obtain the features including GLCM_Contrast, GLCM_Correlation, GLCM_Dissimilarity, GLCM_Entropy, GLCM_Homogeneity, GLCM_Mean, and GLCM_Std values in this study. The image homogeneity is measured by GLCM homogeneity, which presented the similarity in gray level between nearby pixels. GLCM Contrast is a metric to show the number of regional differences in the image opposite GLCM homogeneity. GLCM dissimilarity receives high values when the local areas have high contrast, and it rises linearly opposite to Contrast. GLCM entropy is negatively correlated with energy, and it measures the disorder of an image [45]. The frequency of occurrence of a pixel value in combination with a specific adjacent average pixel value is used to measure GLCM Mean. GLCM standard deviation is not similar to the simple standard deviation; it is based on the GLCM, and deals mainly with the mixtures of neighbor and reference pixels, and GLCM correlation estimates the linear relationship between surrounding pixels’ grey levels. These features, along with the spectral features, are fed into the model to classify the image accurately. To obtain samples for the training dataset and create polygons for each land cover class, we manually analyzed the main images in ArcGIS 10.6. Table 1 shows the total number of samples obtained in the region of research for training and test. A total of 81,831 samples were chosen, and 60%, 15%, and 25% of them were randomly split into training, validation and testing sets, respectively. We then employed a data normalization method to speed up the gradient descent optimization and activation function process [46]. Thus, to avoid anomalous gradients, we performed a z-score normalization method to the pixel values of the aerial imagery (Equation (1)).

(1) $$X^{\prime }=\frac{(X/\max)-\mu }{\sigma }$$

where, the highest pixel value of the image is defined as max , the standard deviation and mean of X/max are defined as σ and μ , respectively, and the normalized data is denoted as $X^{\prime }$ .

### 2.2. DNN Architecture

**Figure 3:**
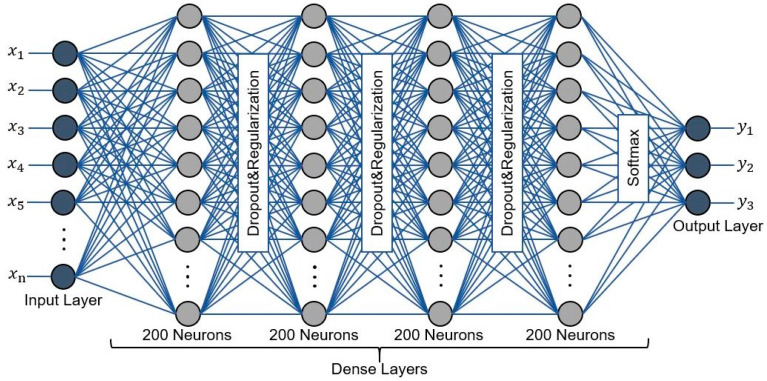
The structure of the proposed DNN network.

Our DNN model is a series of dense layers (fully connected) that take every spectral and textural factor’s attribute as input and classify them to build the classification map, as illustrated in Figure 3. The output values are computed successively along with the network layers when the input features are fed into the DNN. The weighted sum is produced at every layer by multiplication of the weight vector for every unit in the current hidden layer by the input vector, which includes every unit’s output values from the prior layer. An input layer $L_{in}$ , an output layer $L_{out}$ , and H hidden layers $L_{h}(h\in \left\{1,2,\ldots ,H\right\})$ between input and output layers generate the basic DNN architecture. Every hidden layer $L_{h}$ consists of a collection of units that can be structured as a vector $a_{h}\in R^{\left|L_{h}\right|}$ , with $\left|L_{h}\right|$ denoting the number of units in $L_{h}$ . After that, an activation function f(·) , a bias vector $b_{h}\in R^{\left|L_{h}\right|}$ , and a weight matrix $W_{h}\in R^{\left|L_{h1}\right|*\left|L_{h}\right|}$ can then be used to parameterize every hidden layer $L_{h}$ . The units in $L_{h}$ can be computed more completely by

(2) $$a_{h}=f(W_{h}^{T}a_{h-1}+b_{h})$$

where, the units $a_{0}$ in the input layer $L_{0}$ come from a compound’s features vector and h=1,2,…,H . The rectified linear unit (ReLU) function was employed as the activation function in this study, and it is calculated as

(3) f(x)=max(0,x)

Because of the lack of gradient vanishing impact, great computing efficiency, and sparsity property during back-propagation training, ReLU is a popular option of activation function in deep learning [47,48]. We utilized the Softmax function for the output layer $L_{out}$ to predict class possibilities after calculating $a_{H}$ for the last hidden layer. Moreover, to train the suggested network for classification, we used the categorical cross-entropy (CCE) loss function [49]. The loss function for CCE is calculated as follows:

(4) $$L_{CCE}(g,f(I),\delta_{1})=\sum_{i=1}^{S}\sum_{j=1}^{P}\sum_{c=1}^{C}-1(g_{i}^{j}=C)\log l_{c}(I_{i}^{j})$$

where, the number of classes is C , the ground truth label is $g_{i}^{j}$ , the output of the last dense layer at the pixel $I_{i}^{j}$ is $f(I_{i}^{j})$ , the network parameters are $\delta_{1}$ , the size of the batch is S , the jth pixel in the ith patch is $I_{i}^{j}$ , the number of pixels in each patch is P , every class possibility of the pixel $I_{i}^{j}$ is $l_{c}(I_{i}^{j})$ that is denoted as

(5) $$l_{c}(I_{i}^{j})=\frac{\exp(f_{c}(I_{i}^{j}))}{\sum_{l=1}^{c}exp(f_{l}(I_{i}^{j}))}.$$

The suggested method’s architecture is based on a multilayer feed-forward neural network that is organized as below: (1) the input layer has several features representing the class descriptors. (2) There are seven hidden layers. Four layers are dense layers, and three regularization and dropout layers were inserted between them to avoid the over-fitting problem. (3) To influence the collection of obtained features from the final hidden layer to the appropriate class, the Softmax classifier was applied in the last layer. (4) The input layer was regulated based on the kernel regularization method, and the ReLU activation function was also implemented to avoid saturation during the learning process. Moreover, to maintain as many features as feasible, we deleted the pooling operation. This is due to the fact that we want to capture more contextual information and identify fine details [50]. We used Python and the Keras with TensorFlow as a backend to apply the entire process of the suggested model for vegetation mapping from aerial data.

### 2.3. SHAP Approach

SHAP is a game-theoretic way to describing the model’s performance [51]. SHAP employs an additive feature imputation approach, in which an output model is specified as a linear addition of input parameters to build an interpretable method. To explain predictions from the models, a few strategies have been proposed. Under the domain of additive feature attribution approaches, the SHAP structure [39] collects earlier suggested explanation techniques like DeepLIFT [52] and LIME [53]. For an original method f(x) , the explanation approach $g(x^{\prime })$ with streamlined input $x^{\prime }$ can be defined as $f(x)=g(x^{\prime })=\phi_{0}+\sum_{i=1}^{M}\phi_{i}{x^{\prime }}_{i}$ if we presume a method with input parameters $x=(x_{1},x_{2},\ldots ,x_{p})$ , where the number of input parameters is defined as p , the consistent value when all inputs are missing is defined as $\phi_{0}$ , and the number of input features is denoted as M . SHAP has a strong theoretical foundation, which is advantageous in supervised scenarios. By allocating a significance value (SHAP value) to any variable that meets the following criteria, it explains a particular prediction using Shapley values. The requirements [54] include (1) consistency—if we change a model to make it more dependent on a particular component, the relevance of that component should not diminish, notwithstanding the relevance of other variables; (2) missingness—components that aren’t present in the primary input must be ignored; (3) local accuracy—the explanation method has to at least in accordance with the main model’s output. Therefore, SHAP can describe both global and local methods effectively. To develop an interpretable model, which takes into consideration the closeness to the instance, a local model employs a minimal set of background from the data [53]. There are various approaches like Tree SHAP, Deep SHAP, and Kernel SHAP to approximate SHAP values [39]. Kernel SHAP, a model-agnostic estimation, constructs a local explanatory model using Shapley values and linear LIME [53]. Thus, in this work, Kernel SHAP was used because it gives better precise estimations with lower model assessments compared to the other sampling-based estimates.

### 2.4. Assessment Measures

The proposed method’s efficacy was tested using measurement variables such as precision, recall, F1 score, and overall accuracy (OA), as indicated in Equations (6)–(9). The recall is defined as the percentage of accurately detected pixels among all actual pixels. The proportion of accurately categorized pixels among all predicted pixels is known as precision. The F1 score comprises recall and precision, and overall accuracy (OA) is measured as adding the ratio of correctly identified pixels and dividing by the total number of pixels [50].

(6) $$\mathrm{Recall}\;=\;\;\;\frac{TP}{TP+FN}$$

(7) $$\mathrm{Precision}=\frac{TP}{TP+FP}$$

(8) $$F1\;\mathrm{score}=\frac{2\times \mathrm{Precision}\times \mathrm{Recall}}{\mathrm{Precision}+\mathrm{Recall}}$$

(9) $$\mathrm{OA}=\frac{TP+TN}{N}$$

where, *FP* denotes false-positive pixels, *FN* denotes false-negative pixels, *TP* denotes true-positive pixels, *TN* denotes true-negative pixels, and *N* denotes the number of pixels.

### 2.5. DDN Implementation

To analyze the influence of low and high-contributed features on vegetation classification, we first fed all the variables into the model and obtained the classification results. Subsequently, we just fed the low-contributed variables and high-contributed variables obtained by the SHAP method into the model and achieved the classification results. In the following, the features explanations achieved by the SHAP approach are firstly presented and then both quantitative and qualitative results achieved by the DNN model for vegetation mapping based on all variables and high-contributed variables is discussed.

## 3. Results

**Figure 4:**
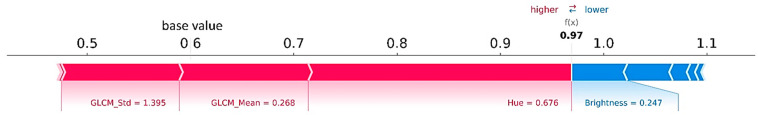
Local interpretation using SHAP force plot for the initial prediction produced by the DNN model. The outcome is pushed lower by blue feature attributions, whereas the results are pushed higher than the “base value” by red feature attributions.

In this section, we graphically presented the features explanations achieved by the SHAP approach. A bar plot, which illustrates the global relevance of the features, or a partial dependence plot, which shows the impact of shifting an individual characteristic, is commonly used to depict the influence of the features in a model [55]. However, other forms of visualizations are possible since SHAP values are outcomes of individualized attributes that are specific to every prediction. Because SHAP dependence graphs reflect the impacts of feature relationships better than partial dependence plots, they are a good alternative to partial dependence plots. To highlight the global significance, SHAP summary graphs replace usual bar plots, and local explanations are displayed using force plots [55]. For example, Figure 4 shows an explanation of a prediction (vegetation class) obtained with a DNN model based on a force plot. The explanation demonstrates how multiple factors work together to push the output of the method from the “base” to the “predicted” value for the initial prediction. The prediction process begins with the baseline. The average of all predictions is the baseline for Shapley values. Each Shapley value is represented by an arrow in the plot, indicating whether the prediction should be increased or decreased. Features that cause a high classification value are represented in red, while those that cause a lower value are indicated in blue. For example, for the vegetation class, the sample has a prediction of 0.97, while the baseline is 0.6. The Hue, GLCM_Std, and GLCM_Mean can improve the final output prediction while Brightness can reduce the prediction.

**Figure 5:**
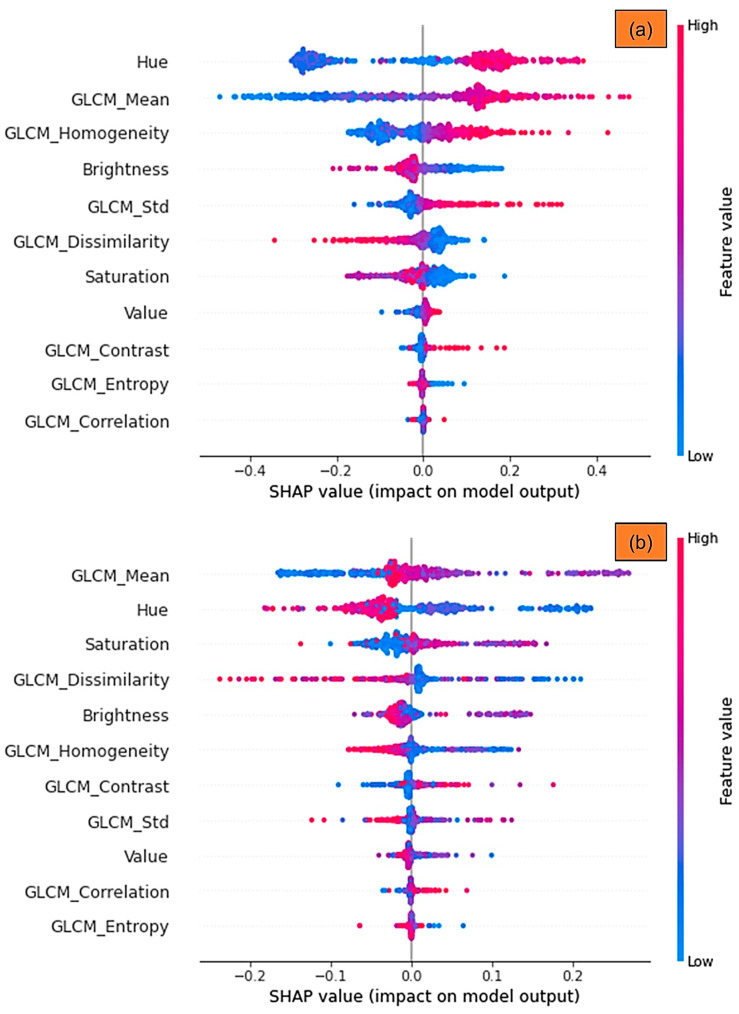
The DNN model’s SHAP summary plot for: (**a**) non-vegetation; and (**b**) vegetation classes. The higher classification is related to the higher SHAP value of a feature.

**Figure 6:**
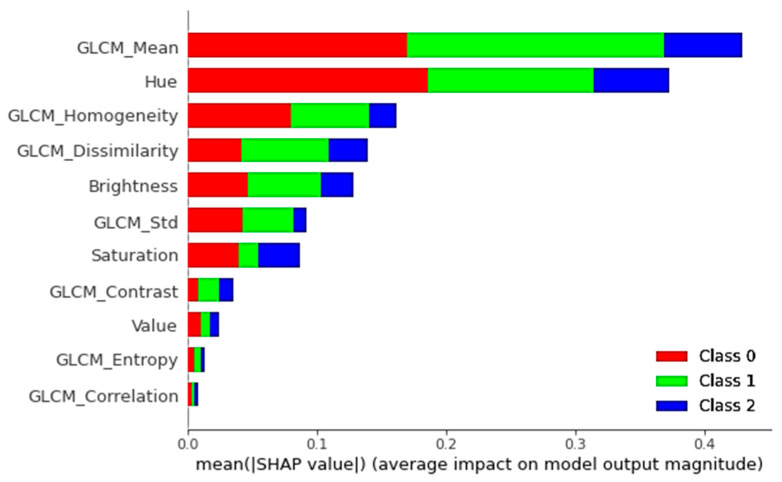
Taking the average absolute value of the SHAP values to measure the relative significance of every feature for every class (e.g., Class 0 is Background, Class 1 is High-Vegetation, and Class 2 is Low-Vegetation).

In addition, we used a summary plot to integrate the feature’s significance with the feature’s impacts. A Shapley value for a feature and a sample is represented by each point on the summary plot. Figure 5a,b, present the summary plots for the non-vegetation and vegetation classes for different labels, respectively. The *x*-axis determines the Shapley value, and the *y*-axis shows the features. The color denotes the feature’s value, which ranges from low to high. We can get a sense of Shapley values dispensation per feature using overlapping points that are jittered in the direction of the *y*-axis. The features are ranked in order of significance [53]. For example, Figure 5a shows that a high degree of Hue, GLCM_Mean and GLCM_Homogeneity contents have a significant and beneficial impact on classification. The red color represents a high, and the *x*-axis represents a positive value. On the opposite, the feature saturation is negatively related to the target factor. Moreover, for the normal bar graph, as shown in Figure 6, we could take the average absolute value of SHAP values to every feature. The SHAP values on the *x*-axis represent the magnitude of the difference in log-odds. In Figure 5, we can see how SHAP values are allocated to the features globally. All features are continual in this scenario, and their average influence on classification is vertically arranged. We can observe how GLCM_Mean has the most significant power on the classification of Class 1 that is High-Vegetation class, while Hue showed a high effect on the Class 0 (Background) classification. In the same manner, high values of GLCM_Homogeniety and GLCM_Dissimilarity are similarly crucial in the classification of non-vegetation and vegetation classes.

**Figure 7:**
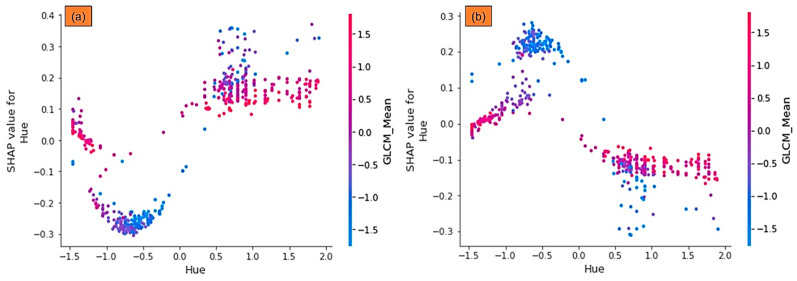
SHAP dependence plots for the DNN method regarding to Hue and GLCM_Mean for non-vegetation (**a**) and vegetation (**b**) classes.

When the value of the features is constant, the SHAP dependence graphs indicate the expected results of a method. In the dependence plots, we can demonstrate how the model is dependent on a feature by illustrating how the model outputs shift when the features vary. For instance, in Figure 7, we plotted the interactions between variables for the non-vegetation class (Background (7a)) and vegetation class (7b) concerning SHAP values to gain a better understanding of their relationships. This is illustrated for Hue and GLCM_Mean in Figure 7, where the colors reflect the SHAP values given to one of them, and x and *y*-axis represent the magnitude of these variables. In fact, the effect of Hue is depicted for variation of GLCM_Mean from −1.5 to 1.5. The high values of the variable GLCM_Mean are represented by red, while the low values are represented by blue. In Figure 7a, the SHAP values for Hue are negative when Hue is less than 0; the SHAP values are extremely low for low Hue and GLCM_Mean, which results in a lower possibility of Background class.

## 4. Discussion

**Figure 8:**
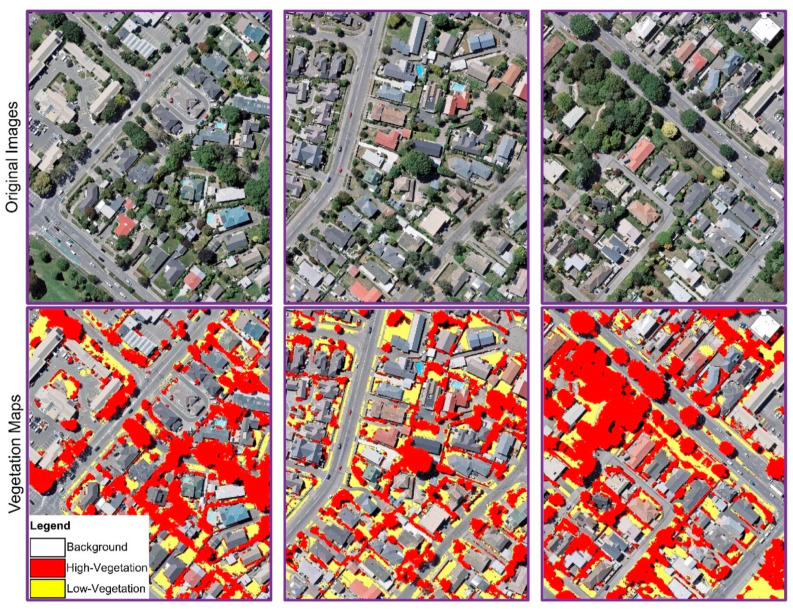
Qualitative outcomes of the presented DNN model for vegetation mapping with high-contributed input features.

**Figure 9:**
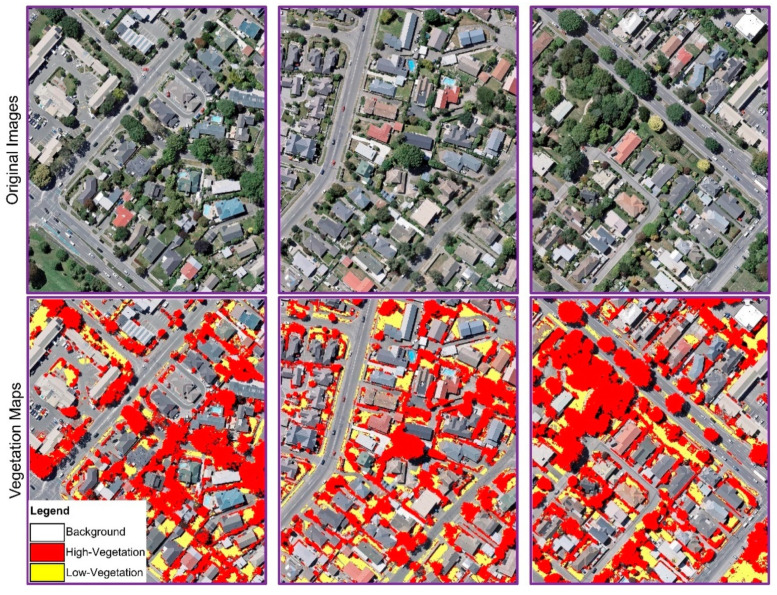
Qualitative outcomes of the presented DNN model for vegetation mapping with all the input features.

Several images with complex backgrounds were used to verify the proposed DNN model for classifying vegetation cover and SHAP method for selecting the appropriate spectral and textural features to see the contribution information of each feature on classification. Based on allocating the SHAP values to every feature and interpreting the effect of the features on the classification in Section 3, we figured out that the features such as Hue, Brightness, GLCM_Dissimilarity, GLCM_Homogeneity, and GLCM_Mean showed high contribution information to the output of the proposed model for classification, while the other features showed low contribution. We showed the qualitative results obtained by the DNN network for the vegetation mapping based on high-ranked features in Figure 8, while the qualitative results for the model with all the input features is depicted in Figure 9. Two rows and three columns separate the sub-figures. The first row shows the main images, while the second row shows the classification results by the DNN method. As seen in Figure 9, the model could not produce high-quality vegetation maps when we use all the features with low contribution features (e.g., GLCM_Correlation, GLCM_Entropy, Value, GLCM_Contrast etc.); the method misclassified pixels (predicted more FPs) which yielded low classification accuracy. For example, in complex backgrounds where obstacles such as buildings, shadows, and other elements obscure vegetation pixels, the model misclassified non-vegetation pixels as vegetation pixels. In contrast, using the high-contributed input features, the suggested DNN technique could get better results and generate high-resolution maps (shown in Figure 8). By looking at the figures, we can see the effect of the high-contributed features on the performance of the DNN model. These features helped the model to identify a smaller number of FP pixels for the classes and classify the images more accurately.

We also generated the assessment metrics to measure the quantitative outputs achieved by our proposed methodology. Table 2 and Table 3 show the average quantitative results for the imagery, with all the features and with high-contributed input features, respectively. As demonstrated in Table 2, the proposed approach achieved classification accuracy of an average OA of 93.11% when we utilized all the input features, while the model achieved an average OA of 94.44% when we used the high-ranked input features for classification (Table 3). In other words, adding high-contributed features to the input improves classification map accuracy, and the method could increase accuracy by 1.33% compared to results obtained with all inputs. In addition to increasing the model’s OA, the combination of high-contributed features also enhanced the accuracy for other metrics of each class label. For instance, the average accuracy of F1 score was attained at 90.66% when the suggested network was applied to images with all features. In contrast, after using the features with high contribution, the model was able to achieve an accuracy of 92.66% for the F1 score. In fact, the proposed network could increase quantitative outcomes by 2%, allowing for more accurate identification of vegetation pixels, even in the presence of complicated areas and large areas of occlusion. The reason is that the proposed DNN model extract deep features from input data in a hierarchical manner and thus improves the classification results.

Moreover, we compared our results with other comparative methods to show the effectiveness of the proposed technique in urban vegetation mapping. For example, Zhang, Feng, and Jiang [21] applied an object-oriented approach that can incorporate spatial and spectral information of objects in the classification procedure for vegetation mapping from IKONOS images. They achieved an average OA of 84.18%, which has a 10.26% difference compared to our method. In another work, Feng, Liu, and Gong [1] implemented a hybrid approach that combined RF and texture analysis to distinguish land covers in urban vegetated regions from UAV imagery. An OA of 88.4% was achieved by their method, while it is less than the OA obtained by our method. In [4], XGBoost method was applied for vegetation mapping from MODIS and LANDSAT data for two various cases (New Zealand and the Dzungarian Basin in China). They obtained an average OA of 92.65% that is 1.79% less than the results achieved by our method. By comparing the results achieved with other methods and our proposed method, it is shown that the DNN technique demonstrated efficiency in vegetation mapping from aerial images.

## 5. Conclusions

We used aerial imagery to categorize land-use types and generate urban vegetation maps using a deep neural network. However, to (1) comprehend model decisions, (2) grasp complicated inherent non-linear relations, and (3) determine the model’s suitability for more monitoring and evaluation, it is critical to comprehend why a data-driven method makes any classification based on specific input data. Thus, we also used an explanation model called SHAP to interpret the outputs of a DNN model designed for classification and analyze the feature importance. In fact, we applied the SHAP technique to select appropriate features for classification based on allocating SHAP values for every feature of every data point that contributes to the model’s output. We utilized some spectral and textural features as an input for the model to classify the imagery and generate vegetation maps. Based on interpreting the model’s output using SHAP, we realized features such as GLCM_Homogeneity, GLCM_Mean, GLCM_Dissimilarity, Hue, GLCM_Homogeneity, GLCM_Mean, and Brightness presented high contribution information to the output of the proposed model, while GLCM_Correlation, GLCM_Entropy, Value, and GLCM_Contrast showed low contribution. Therefore, we tried to classify the images with those low-ranked and high-ranked features to find the influence of these attributes on the model’s performance. We achieved both visualization and quantitative outcomes, which confirmed that the presented DNN model could enhance the OA accuracy and generate high-resolution vegetation maps using the high-contributed features. This study also showed that the SHAP yielded the features’ importance values and enabled the interpretation of the model and its predictions, which confirm that the method is applicable and appropriate for interpreting the machine learning models. To end this, SHAP allows us to conduct in-depth analysis of the data and leads us in the selecting appropriate features and AI model for vegetation classification. However, the DNN algorithm disregarded the importance between features, which implies we may lose the association between features. Secondly, when training a large dataset, the kernel SHAP approach is slow. Finally, the work’s interpreters aided us in breaking open the black box, but further research is needed to compare and evaluate existing interpretation approaches.

## Figures and Tables

**Table 1:** The size of training and testing sets employed in this work counted at polygon and pixel-level.

Class	Polygons	Training Samples (Pixels)	Validation Samples (Pixels)	Testing Samples (Pixels)	Legend
Background	254	34,209	5684	16,240	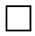
High-Vegetation	198	11,611	2049	5854	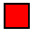
Low-Vegetation	160	3085	544	2555	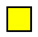

**Table 2:** Quantitative results attained by the model with all the input features.

	Precision	Recall	F1_score	OA
Background	0.94	0.98	0.96	93.11
High-vegetation	0.93	0.83	0.88	
Low-vegetation	0.90	0.87	0.88	

**Table 3:** Quantitative results attained by the model with high-contributed features.

	Precision	Recall	F1_score	OA
Background	0.95	0.99	0.97	**94.44**
High-vegetation	0.95	0.84	0.89	
Low-vegetation	0.90	0.94	0.92	

## Data Availability

The link to download the Massachusetts dataset can be found in the online version, at https://www.airs-dataset.com/ (accessed on 18 June 2021).
